# Supplementary material for: Dynamic emotional states shape the episodic structure of memory
Source: Nat Commun. 2023 Oct 17;14:6533. doi: 10.1038/s41467-023-42241-2 (PMC10582075; doi:10.1038/s41467-023-42241-2)
Supplement: Supplementary file 1 — Supplementary Information [file 41467_2023_42241_MOESM1_ESM.pdf]

Dynamic emotional states shape the episodic structure of memory

Mason McClay[1], Matthew E. Sachs[2, 3], & David Clewett[1*]

[1]University of California, Los Angeles, Department of Psychology, Los Angeles, CA, USA
[2]Columbia University, Department of Psychology, New York City, NY, USA
[3]Columbia University, Center for Science and Society, New York City, NY, USA

*Correspondence: david.clewett@psych.ucla.edu

SUPPLEMENTARY INFORMATION

piece number	Clip1 emotion	Clip1 sec	Transition1 sec	Clip2 emo	Clip2 sec	Transition2 sec	Clip3 emotion	Clip3 sec	total seconds
2	anxious	30.5	9	calm	51	9	joyous	30.5	130
3	joyous	41	9	anxious	30	6	sad	44	130
4	sad	42.5	9	joyous	27	9	calm	42.5	130
5	calm	32	9	sad	48	9	anxious	32	130
6	dreamy	32	9	sad	48	9	anxious	32	130
6	anxious	32	9	calm	48	6	joyous	35	130
7	joyous	29	12	anxious	48	9	sad	32	130
8	dreamy	29	9	calm	54	9	joyous	29	130
9	joyous	32	9	sad	48	9	anxious	32	130
10	anxious	29	9	joyous	54	6	calm	32	130
11	calm	27.5	9	anxious	57	9	sad	27.5	130
12	joyous	32	9	sad	48	9	anxious	32	130

Supplemental Table 1. Composer emotional events and transition labels. Composers created different emotional songs, which were spliced to create 12 songs each containing 3 emotional events and 2 transitions with *a priori* event timing. Eleven songs were used in the present study, including one practice song (piece number 1: "dreamy_calm_joyous"), and excluding one song (piece number 6: "dreamy_sad_anxious"). All songs began five seconds before and ended five seconds after the encoded lists, thereby resulting in 120-second-long emotional contexts during encoding. Three emotional events, or segments, were included in each song and were used to induce two musically-evoked emotional event boundaries per list.

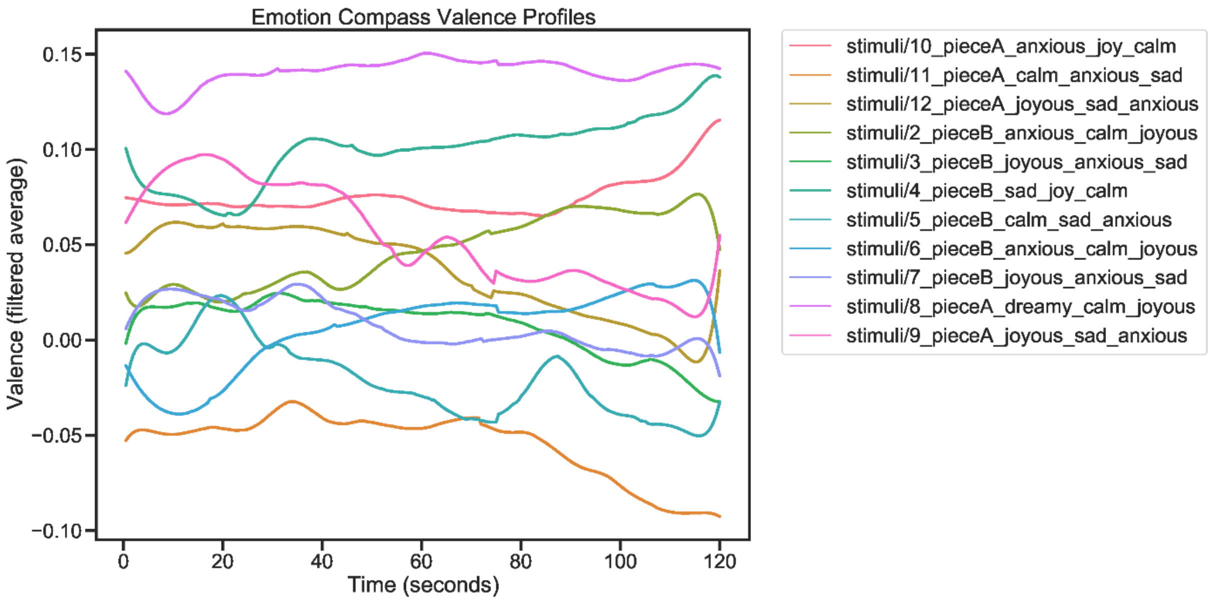

Supplemental Figure 1. Musical valence profiles from the *Emotion Compass*. Song-wise temporal profiles for *Emotion Compass*-extracted valence. Valence scores across the entire song was filtered, cleaned for poor performers, and concatenated across remaining participants (n=65). Profile intercepts and means were not corrected. Any difference in intercept and means were disregarded for Day 1 analyses, which were concerned with change-point locations and ratings-difference scores within-song. All analyses include random intercept for song identity.

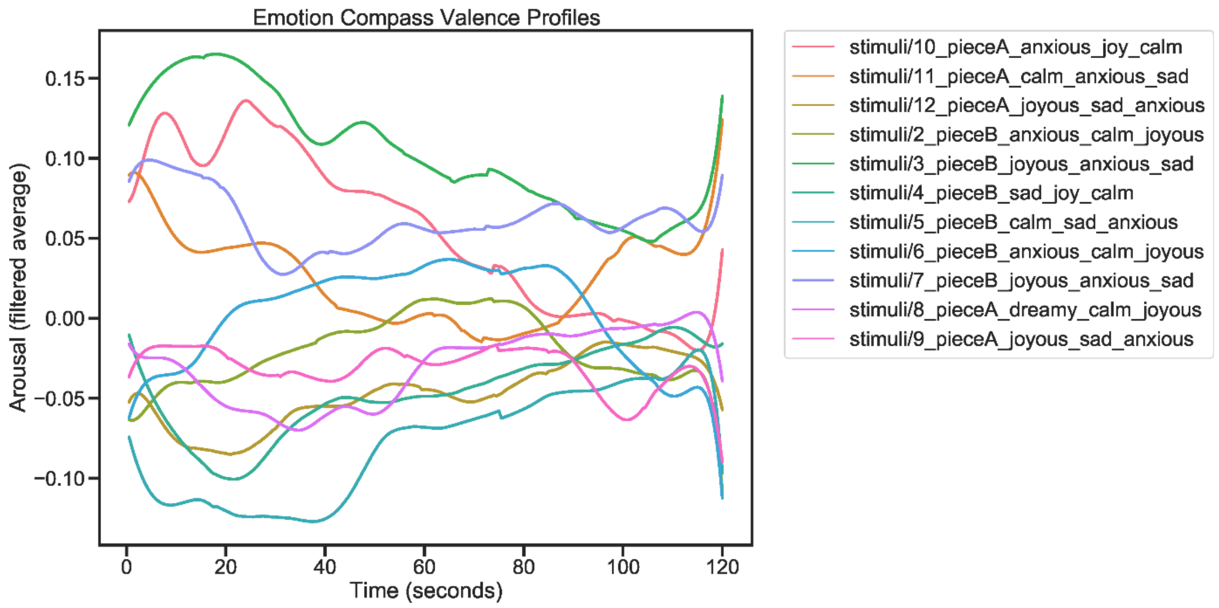

Supplemental Figure 2. Musical arousal profiles from the *Emotion Compass*. Song-wise temporal profiles for *Emotion Compass*-extracted arousal. Arousal scores across the entire song were filtered, cleaned for poor performers, and concatenated across remaining participants (n=65). Profile intercepts and means were not corrected. Any difference in intercept and means were disregarded for Day 1 analyses, which were concerned with change-point locations and ratings-difference scores within-song. All analyses include random intercept for song identity.

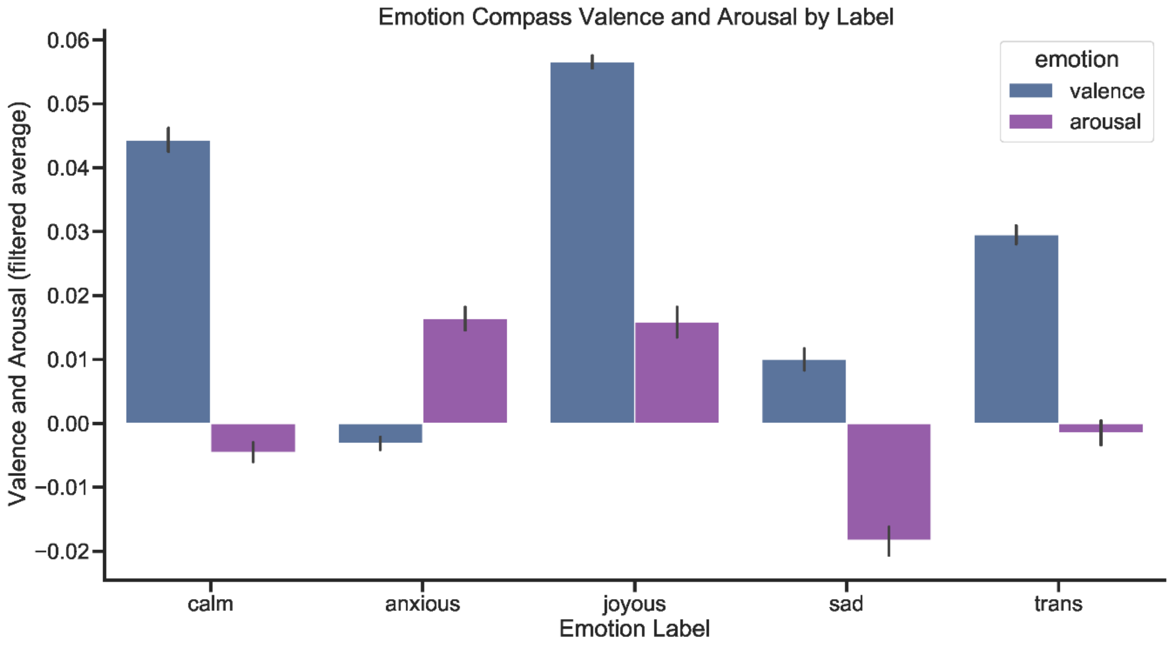

Supplemental Figure 3. *Emotion Compass* subjective valence and arousal rating by composer-defined emotion categories. Composer-labeled emotions were validated by *Emotion Compass*-extracted valence and arousal averages. Positive emotion labels (calm; joy) were much higher in valence than negative emotion labels (sad; anxious). Similarly, high arousal emotion labels (joy; anxious), were much higher in arousal than low arousal emotion labels (calm; sad). Interestingly, transition periods valence and arousal means were similar to the overall mean of valence and arousal, suggesting that transitions were distinct from any specific emotional state. Error bars = 95% bootstrapped CI.

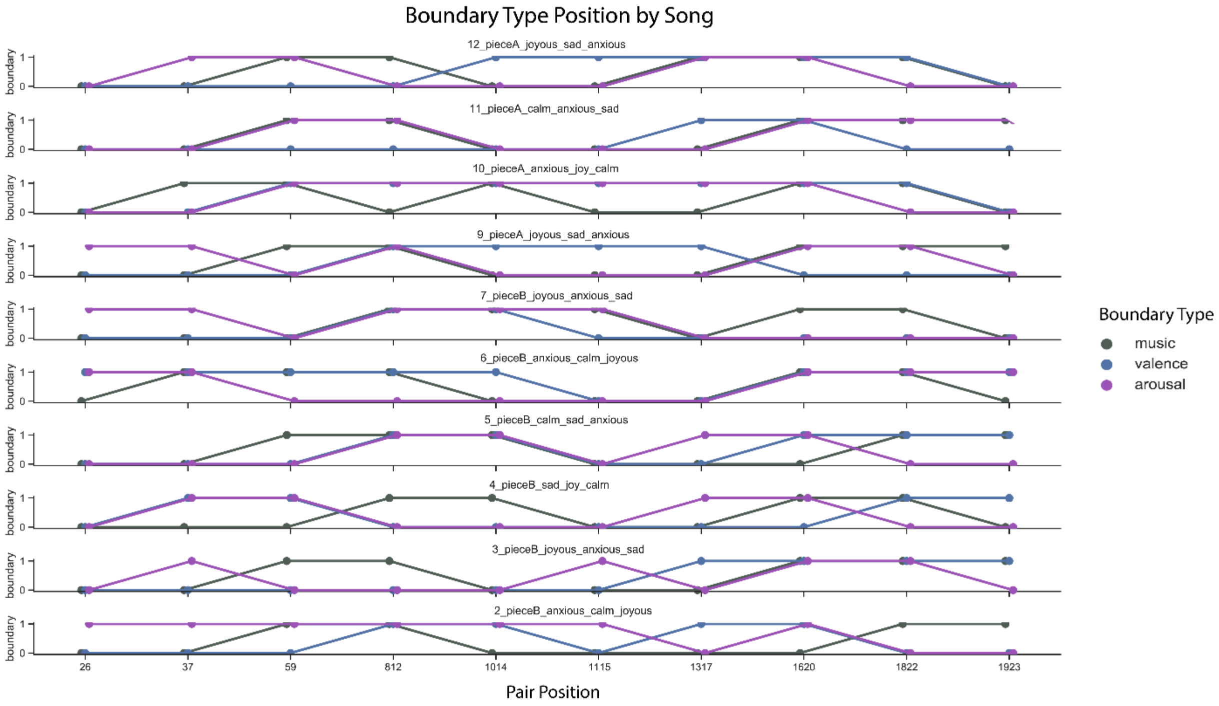

Supplemental Figure 4. Boundary types at different to-be-tested item pair locations during encoding. Boundary types (musical, valence, and arousal) varied in position across all songs.

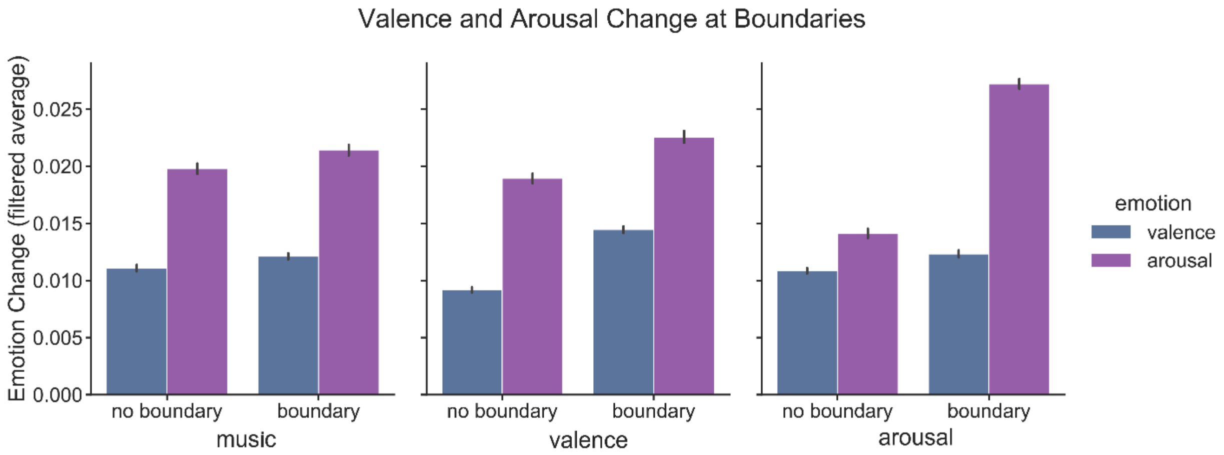

Supplemental Figure 5. Changes in valence and arousal between to-be-tested item pairs at different boundary types. Change in valence and arousal is greater for item pairs spanning valence and arousal change-points, respectively, compared to item pairs not spanning valence and arousal change-points. The overall pattern indicates that valence and arousal change is greatest at their respective boundaries compared to non-boundaries. Error bars = 95% bootstrapped CI. Blue = valence change; purple = arousal change.

Model Key

D = Subjective temporal distance
T = Temporal order/ Temporal displacement
A = Arousal boundaries
V = Valence boundaries
M = Music boundaries
AC = Arousal absolute change
VC = Valence absolute change
AS = Arousal signed change
VS = Valence signed change
R = Recognition
CP = Changepoint

Model	Predictors	R2_conditional	R2_marginal	RMSE	Sigma	AIC_wt	AICc_wt	BIC_wt
mDA	arousal boundaries	NA	0.000	0.738	0.742	0.000	0.000	0.000
mD	base model	NA	0.000	0.738	0.742	0.000	0.000	0.016
mDM	musical boundaries	NA	0.001	0.738	0.742	0.001	0.001	0.007
mDV	musical, valence, arousal boundaries	NA	0.002	0.737	0.741	0.086	0.086	0.797
mDMVA	valence boundaries	NA	0.003	0.737	0.741	0.280	0.279	0.002
mDMV	musical and valence boundaries	NA	0.003	0.737	0.741	0.633	0.633	0.178

Supplemental Table 2. Model fit statistics for subjective temporal distance boundary hierarchical linear models.

Model	Predictors	R2_conditional	R2_marginal	RMSE	Sigma	AIC_wt	AICc_wt	BIC_wt
mTA	arousal boundaries	0.15335709	0.000	0.412	1.000	0.000	0.000	0.000
mT	base model	NA	0.000	0.412	1.000	0.000	0.000	0.000
mTM	musical boundaries	0.156016941	0.003	0.412	1.000	0.000	0.000	0.000
mTMVA	musical, valence, arousal boundaries	NA	0.010	0.411	1.000	0.449	0.449	0.019
mTV	valence boundaries	NA	0.007	0.412	1.000	0.005	0.005	0.233
mTMV	musical and valence boundaries	NA	0.010	0.411	1.000	0.546	0.546	0.748

Supplemental Table 3. Model fit statistics for temporal order boundary hierarchical linear models.

Name	Model	R2_conditional	R2_marginal	RMSE	Sigma	AIC_wt	AICc_wt	BIC_wt
mD	base model	NA	0.000	0.741	0.744	0.508	0.509	0.971
mDVC	valence (absolute change)	NA	0.000	0.741	0.744	0.492	0.491	0.029

Supplemental Table 4. Model fit statistics for subjective temporal distance predicted by absolute change in valence.

Model	Predictors	R2_conditional	R2_marginal	RMSE	Sigma	AIC_wt	AICc_wt	BIC_wt
mT	base model	NA	0.000	0.413	1.000	0.716	0.716	0.988
mTVC	valence (absolute change)	NA	0.000	0.413	1.000	0.284	0.284	0.012

Supplemental Table 5. Model fit statistics for temporal order predicted by absolute change in valence.

Name	Model	R2_conditional	R2_marginal	RMSE	Sigma	AIC_wt	AICc_wt	BIC_wt
mD	base model	NA	0.000	0.741	0.744	1.000	0.509	0.971
mDAC	arousal (absolute change)	NA	0.000	0.738	0.741	0.000	0.000	0.000

Supplemental Table 6. Model fit statistics for subjective temporal distance predicted by absolute change in arousal.

Name	Predictors	R2_conditional	R2_marginal	RMSE	Sigma	AIC_wt	AICc_wt	BIC_wt
mT	base model	NA	0.000	0.413	1.000	1.000	1.000	1.000
mTAC	arousal (absolute change)	NA	0.000	0.413	1.000	0.000	0.000	0.000

Supplemental Table 7. Model fit statistics for temporal order predicted by absolute change in arousal.

Name	Model	R2_conditional	R2_marginal	RMSE	Sigma	AIC_wt	AICc_wt	BIC_wt
mD	base model	NA	0.000	0.741	0.744	0.091	0.091	0.763
mDVS	valence (signed change)	NA	0.000	0.740	0.744	0.909	0.237	0.237

Supplemental Table 8. Model fit statistics for subjective temporal distance predicted by signed change in valence.

Name	Model	R2_conditional	R2_marginal	RMSE	Sigma	AIC_wt	AICc_wt	BIC_wt
mD	base model	NA	0.000	0.736	0.741	0.728	0.729	0.987
mDAS	arousal (signed change)	NA	0.000	0.736	0.741	0.272	0.271	0.013

Supplemental Table 9. Model fit statistics for subjective temporal distance predicted by signed change in arousal.

Name	Model	R2_conditional	R2_marginal	RMSE	Sigma	AIC_wt	AICc_wt	BIC_wt
mT	base model	NA	0.000	0.413	1.000	0.251	0.251	0.915
mTVS	valence (signed change)	0.155	0.001	0.412	1.000	0.749	0.749	0.085

Supplemental Table 10. Model fit statistics for temporal order predicted by signed change in valence.

Name	Model	R2_conditional	R2_marginal	RMSE	Sigma	AIC_wt	AICc_wt	BIC_wt
mT	base model	0.155	0.000	0.410	1.000	0.589	0.590	0.976
mTAS	arousal (signed change)	0.156	0.000	0.410	1.000	0.411	0.410	0.024

Supplemental Table 11. Model fit statistics for temporal order predicted by signed change in arousal.

Name	Model	R2_conditional	R2_marginal	RMSE	Sigma	AIC_wt	AICc_wt	BIC_wt
mR	base model	0.249	0.000	0.444	1.000	0.203	0.204	1.000
mRVA	arousal * valence	0.249	0.000	0.444	1.000	0.797	0.796	0.000

Supplemental Table 12. Model fit statistics for delayed recognition predicted by arousal and valence.

Name	Model	R2_conditional	R2_marginal	RMSE	Sigma	AIC_wt	AICc_wt	BIC_wt
mS	base model	0.023	0.000	4.668	4.676	0.000	0.000	0.000
mSVA	arousal * valence	0.059	0.019	4.651	4.660	1.000	1.000	1.000

Supplemental Table 13. Model fit statistics for delayed temporal source by arousal and valence.

Name	Model	R2_conditional	R2_marginal	RMSE	Sigma	AIC_wt	AICc_wt	BIC_wt
mR	base model	0.249	0.000	0.444	1.000	0.004	0.004	0.162
mRCP	change point type	0.250	0.001	0.443	1.000	0.996	0.996	0.838

Supplemental Table 14. Model fit statistics for delayed recognition predicted by change-point type.

Name	Model	R2_conditional	R2_marginal	RMSE	Sigma	AIC_wt	AICc_wt	BIC_wt
mT	base model	0.023	0.000	0.987	4.676	0.000	0.000	0.000
mTCP	change point type	0.034	0.010	0.981	4.660	1.000	1.000	1.000

Supplemental Table 15. Model fit statistics for delayed temporal source predicted by change-point type.

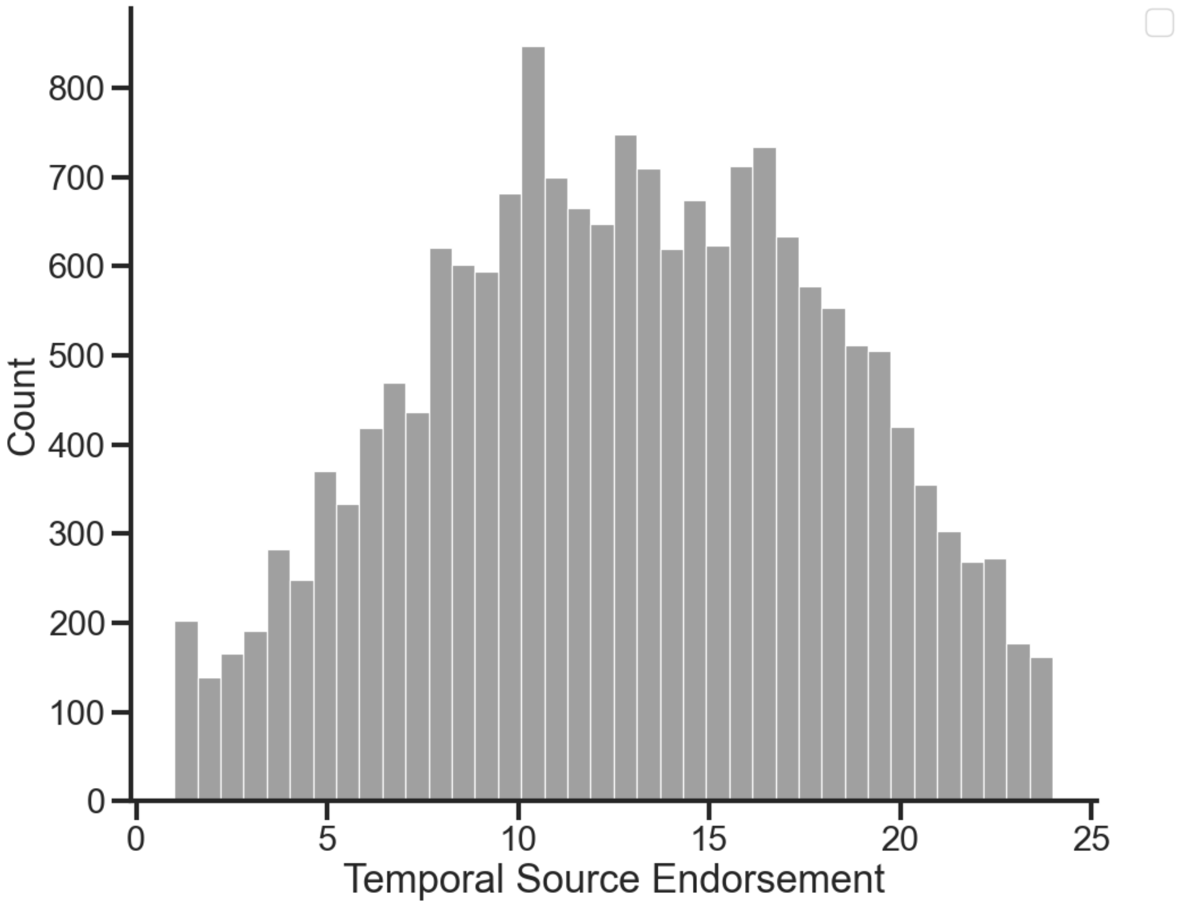

Supplemental Figure 6. Temporal source endorsement histogram by actual item position in a study list. Temporal source endorsement histogram indicates that the frequency of endorsing positions in the middle of the temporal slider was higher than endorsements of positions earlier or later in the list.

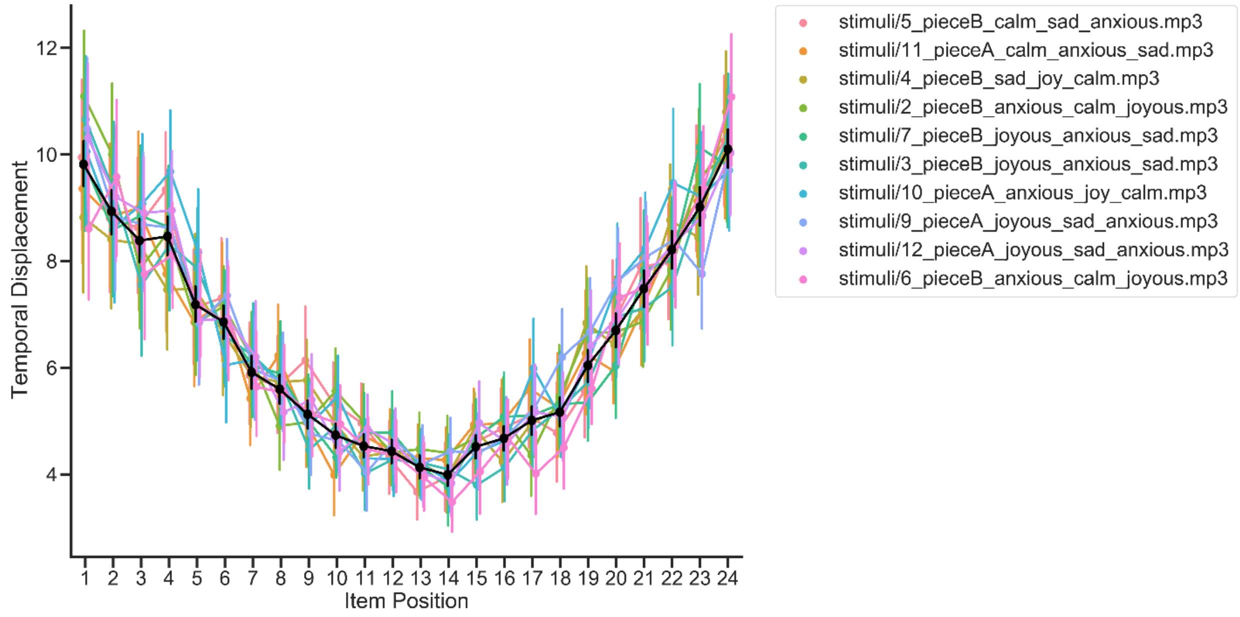

Supplemental Figure 7. Temporal displacement by item position within a study list across songs. Temporal source displacement scores followed a u-shaped curve, suggesting that participants had more accurate temporal judgments for items in the middle versus the beginning or end of the lists. This pattern of results may be a function of the distribution of responses being biased towards positions in the middle of the temporal slider (see **Supplementary Figure 6** above). However, this pattern of results did not confound effect of interest (i.e., temporal displacement for boundary vs non-boundary items; see **Supplementary Figure 9**). Colored bars refer to the type of emotional song (different combination/order of emotion segments). Black dots indicate means collapsed across songs. Error bars = 95% bootstrapped CI.

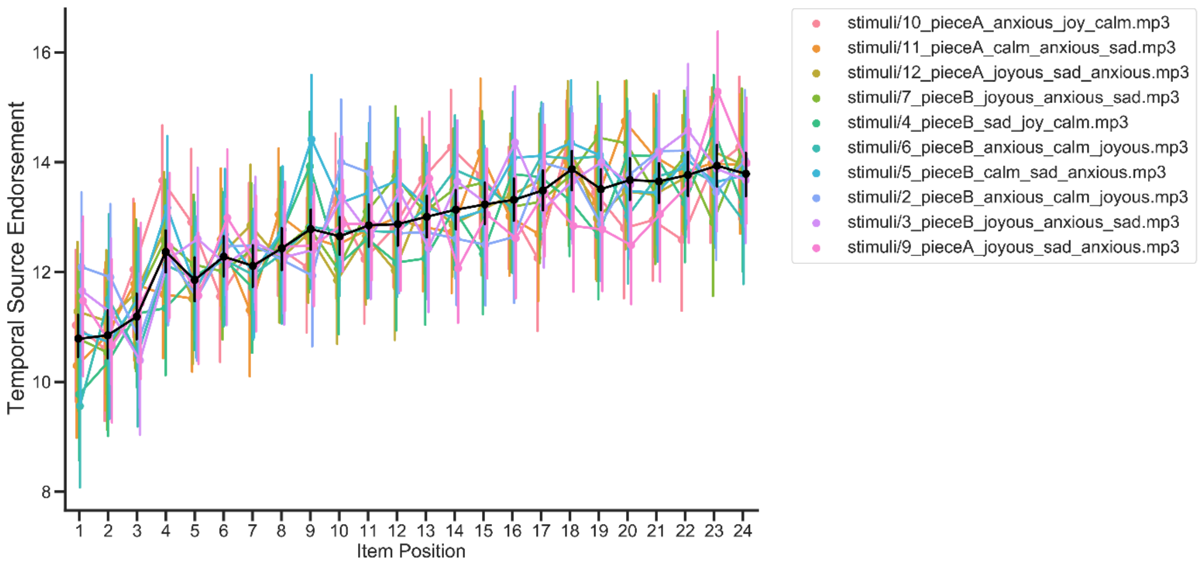

Supplemental Figure 8. Temporal source endorsement by actual item position in a study list. Endorsement of temporal source position by actual item position indicates that endorsement is linearly related to item position during encoding. This suggests that participants were able to accurately encode an item's temporal index from encoding. Colored bars refer to the type of emotional song (different combination/order of emotion segments). Black dots indicate means collapsed across songs. Error bars = 95% bootstrapped CI.

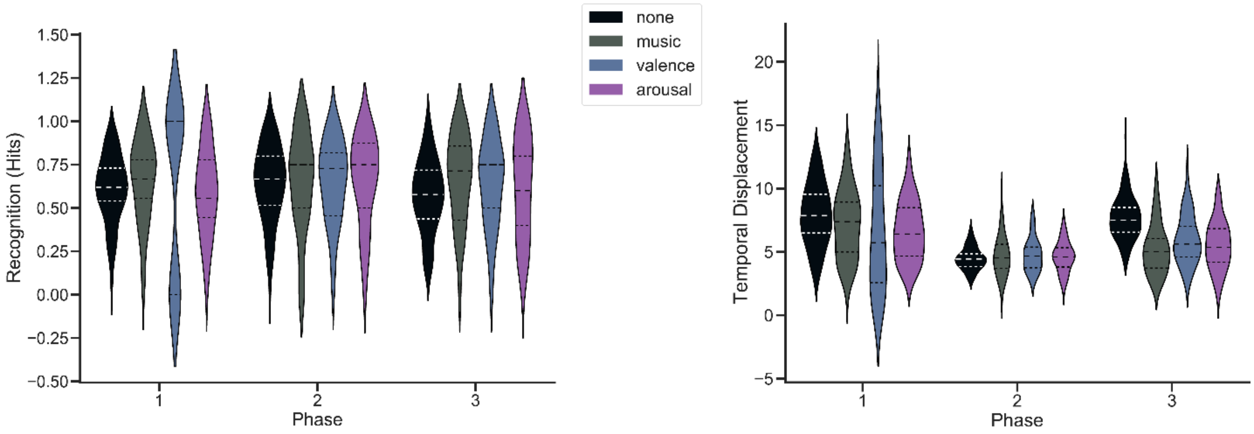

Supplemental Figure 9. Day 2 memory for boundary type by phase of song (early, middle, late). Breakdown of delayed item recognition and temporal displacement memory for boundary types by encoding phase (early: item positions 1-8; middle: item positions 9-16; late: item positions 17-24) shows that effects of enhanced memory for boundaries were not driven by the proximity of boundary items to the center of the song. Neither item recognition nor temporal source memory meaningfully differed for items encountered at boundaries versus non-boundaries within the middle of the songs, but did differ for items encountered at the beginning and end of songs. As expected from the participants' endorsement bias towards the middle of the slider, overall memory was better for items encountered in the middle of the song for all boundary types, including non-boundary items. Violin plots with interquartile ranges represented by dashed lines. *N* = 73 participants.
