# Supplementary material for: Dynamic emotional states shape the episodic structure of memory
Source: Nat Commun. 2023 Oct 17;14:6533. doi: 10.1038/s41467-023-42241-2 (PMC10582075; doi:10.1038/s41467-023-42241-2)
Supplement: Supplementary file 3 — Description of Additional Supplementary Files [file 41467_2023_42241_MOESM3_ESM.pdf]

Description of Additional Supplementary Files

File Name: Supplementary Software 1
Description: Code for the experiment (days 1 and 2) and the Emotion Compass. This software includes the source code for all day 1 tasks (musevent_emotion_compass_day_1.js), all day 2 tasks (musevent_day_2.js), and the Emotion Compass (emotion_compass_only_1.js). All experimental software was built in PsychoPy v2022.2.5 and designed for deployment on Pavlovia.org.
