# Supplementary material for: Dynamic emotional states shape the episodic structure of memory
Source: Nat Commun. 2023 Oct 17;14:6533. doi: 10.1038/s41467-023-42241-2 (PMC10582075; doi:10.1038/s41467-023-42241-2)
Supplement: Supplementary file 5 — Reporting Summary [file 41467_2023_42241_MOESM5_ESM.pdf]

nature portfolio

Corresponding author(s): David Clewett, PhD

Last updated by author(s): 2023/09/06

Reporting Summary

Nature Portfolio wishes to improve the reproducibility of the work that we publish. This form provides structure for consistency and transparency in reporting. For further information on Nature Portfolio policies, see our Editorial Policies and the Editorial Policy Checklist.

Statistics

For all statistical analyses, confirm that the following items are present in the figure legend, table legend, main text, or Methods section.

n/a	Confirmed	
☐	☒	The exact sample size (n) for each experimental group/condition, given as a discrete number and unit of measurement
☐	☒	A statement on whether measurements were taken from distinct samples or whether the same sample was measured repeatedly
☐	☒	The statistical test(s) used AND whether they are one- or two-sided<br>*Only common tests should be described solely by name; describe more complex techniques in the Methods section.*
☐	☒	A description of all covariates tested
☐	☒	A description of any assumptions or corrections, such as tests of normality and adjustment for multiple comparisons
☐	☒	A full description of the statistical parameters including central tendency (e.g. means) or other basic estimates (e.g. regression coefficient) AND variation (e.g. standard deviation) or associated estimates of uncertainty (e.g. confidence intervals)
☐	☒	For null hypothesis testing, the test statistic (e.g. F, t, r) with confidence intervals, effect sizes, degrees of freedom and P value noted<br>*Give P values as exact values whenever suitable.*
☒	☐	For Bayesian analysis, information on the choice of priors and Markov chain Monte Carlo settings
☐	☒	For hierarchical and complex designs, identification of the appropriate level for tests and full reporting of outcomes
☐	☒	Estimates of effect sizes (e.g. Cohen's d, Pearson's r), indicating how they were calculated

Our web collection on statistics for biologists contains articles on many of the points above.

Software and code

Policy information about availability of computer code

Data collection	Open source and custom Python (v 3.11.0) code was used to build the experiment and collect the data in this study. PsychoPy code used to build the experiment are available on the first author's OSF page (https://doi.org/10.17605/osf.io/s8g5n).
Data analysis	Open source and custom Python (v 3.11.0) code as well as R (v 4.2) code was used in cleaning and analyzing the data in this study. For mixed-effects modeling analysis, we used the lme4 package (https://cran.r-project.org/web/packages/lme4/index.html). For change-point analysis, we used the Ruptures package in Python 3 (https://pypi.org/project/ruptures/). The Python and R code used for data processing and analysis are available on the first author's OSF page (https://doi.org/10.17605/osf.io/s8g5n).

For manuscripts utilizing custom algorithms or software that are central to the research but not yet described in published literature, software must be made available to editors and reviewers. We strongly encourage code deposition in a community repository (e.g. GitHub). See the Nature Portfolio guidelines for submitting code & software for further information.

Data

Policy information about availability of data

All manuscripts must include a data availability statement. This statement should provide the following information, where applicable:

- Accession codes, unique identifiers, or web links for publicly available datasets
- A description of any restrictions on data availability
- For clinical datasets or third party data, please ensure that the statement adheres to our policy

Source data associated with the figures, the raw data generated in this study, and all music and image stimuli have been deposited in the first author's OSF profile (https://doi.org/10.17605/osf.io/s8g5n). The image stimuli were selected from the DinoLab Object Database (https://mariamh.shinyapps.io/dinolabobjects).

Research involving human participants, their data, or biological material

Policy information about studies with human participants or human data. See also policy information about sex, gender (identity/presentation), and sexual orientation and race, ethnicity and racism.

Reporting on sex and gender	96 participants were recruited in the current study (61 female). Neither sex nor gender were included as fixed effects or covariates in the current analyses because they were not variables of interest in this study. Sex was not included in the study design, and that data was collected via self-report on a demographics questionnaire.
Reporting on race, ethnicity, or other socially relevant groupings	Of 96 participants recruited in the current study, 35 identified as being non-white (18 Asian, 12 Hispanic, 5 Black). Race, ethnicity, or other socially relevant groupings were not included as fixed effects or covariates in the current analyses because they were not variables of interest in this study. Race, ethnicity, or other socially relevant groupings were not included in the study design, and that data was collected via self-report on a demographics questionnaire.
Population characteristics	See above.
Recruitment	All participants were recruited online using Prolific, a website that connects researchers with eligible participants based on a wide range of inclusion criteria. Eligibility criteria included: (1) age of 18 to 35 years old; (2) United States or Canada nationality; (3) English first language; (4) normal or corrected-to-normal vision; (5) no history of serious head injuries (e.g., concussion); (6) previous participation in 50 studies on Prolific; and (6) a minimum 90% approval rating on Prolific (percentage of studies for which the participant has been approved by the experimenter). Selection bias may have included participants who care to perform well on online studies as well as those who enjoy listening to music. However we do not believe this would bias the results in a meaningful way.
Ethics oversight	All experimental protocols were approved by the University of California at Los Angeles Institutional Review Board.

Note that full information on the approval of the study protocol must also be provided in the manuscript.

Field-specific reporting

Please select the one below that is the best fit for your research. If you are not sure, read the appropriate sections before making your selection.

☐ Life sciences ☒ Behavioural & social sciences ☐ Ecological, evolutionary & environmental sciences

For a reference copy of the document with all sections, see nature.com/documents/nr-reporting-summary-flat.pdf

Behavioural & social sciences study design

All studies must disclose on these points even when the disclosure is negative.

Study description	We used a quantitative experimental study design. All participants listened to music that varies across emotional states (within-subject design with emotion factor) while studying lists of images of everyday items. All participants were tested on various features of episodic memory related to the order, subjective temporal relations following music listening. Participants then returned 24-hours later to be tested on item recognition and source memory of the images.
Research sample	Ninety-six healthy young adult participants (mean age 27 yr old, SD 4.9 yr; 61 female) were recruited online using Prolific, a website that connects researchers with eligible participants based on a wide range of inclusion criteria. Eligibility criteria included: (1) age of 18 to 35 years old; (2) United States or Canada nationality; (3) English first language; (4) normal or corrected-to normal vision; (5) no history of serious head injuries (e.g., concussion); (6) previously participation in 50 studies on Prolific; and (6) a minimum 90% approval rating on Prolific (percentage of studies for which the participant has been approved by the experimenter). Our sample is representative of healthy young adults whose native language is English. This sample was chosen to control for comprehension of instructions and to avoid confounds of psychoactive medication or age/brain related changes to cognitive function.
Sampling strategy	Stimulus order was fully randomized for each participant. To estimate the total number of participants needed in this study, we assumed a moderate effect size for a GLM with our predictors of interest (F-squared = .2) based on the effect size derived from a general linear model with two predictors in a similar emotional event boundaryexperiment (boundary arousal and item event position; GLM model comparison: w = .59). This power analysis indicated a sample size of 51. However, we assumed that on line data

	collection would yield more noise in our sample than traditional lab-based memory experiments, so we increased our target sample size to 80.
Data collection	All data were collected online using participants' home computers. Participants were instructed to perform the experiment in a private room and to use headphones to listen to the music stimuli. Participants used their computer cursors to continuously rate their valence and arousal on the Emotion Compass. The experimenter was not physically present since the study was conducted online, thus limiting experimenter bias. Experimenters were not blinded, but this is unnecessary because all conditions (musically-elicited emotional fluctuations) were within-subjects.
Timing	Data were collected online between January 24, 2022 and January 28, 2022.
Data exclusions	Nine participants (n=9) did not complete the Day 1 task. An additional 6 participants (n=6) that completed the Day 1 task did not meet inclusion criterion of 50% accuracy or above on temporal memory performance, leaving eighty-one participants for all Day 1 temporal memory analyses (n=81). Of those eighty-one participants, seventy-two completed all Day 2 memory tasks (n=72) and were included in all Day 2 memory analyses. All exclusion criteria were pre-registered.
Non-participation	Nine participants (n=9) dropped out or did not complete the study. We do not inquire as to why they logged out of the online study.
Randomization	Participants were not allocated into separate groups because all factors were within-subjects in this study.

Reporting for specific materials, systems and methods

We require information from authors about some types of materials, experimental systems and methods used in many studies. Here, indicate whether each material, system or method listed is relevant to your study. If you are not sure if a list item applies to your research, read the appropriate section before selecting a response.

Materials & experimental systems

n/a	Involved in the study	
☒	☐	Antibodies
☒	☐	Eukaryotic cell lines
☒	☐	Palaeontology and archaeology
☒	☐	Animals and other organisms
☒	☐	Clinical data
☒	☐	Dual use research of concern
☒	☐	Plants

Methods

n/a	Involved in the study	
☒	☐	ChIP-seq
☒	☐	Flow cytometry
☒	☐	MRI-based neuroimaging
